# Autosomal recessive long QT syndrome, type 1 in eight families from Saudi Arabia

**DOI:** 10.1002/mgg3.305

**Published:** 2017-06-21

**Authors:** Amnah Y. Bdier, Saleh Al‐Ghamdi, Prashant K. Verma, Khalid Dagriri, Bandar Alshehri, Omamah A. Jiman, Sherif E. Ahmed, Arthur A. M. Wilde, Zahurul A. Bhuiyan, Jumana Y. Al‐Aama

**Affiliations:** ^1^ Department of Biological Sciences Faculty of Science King Abdulaziz University Jeddah Saudi Arabia; ^2^ Princess Al Jawhara Albrahim Center of Excellence in Research of Hereditary Disorders King Abdulaziz University Jeddah Saudi Arabia; ^3^ Department of Genetic Medicine King Abdulaziz University Jeddah Saudi Arabia; ^4^ Department of Cardiology NGH Riyadh Saudi Arabia; ^5^ Department of Pediatric Cardiology Prince Sultan Cardiac Center Riyadh Saudi Arabia; ^6^ Department of Genetics Faculty of Agriculture Ain shams University Cairo Egypt; ^7^ Department of Cardiology Academic Medical Center University of Amsterdam Amsterdam the Netherlands; ^8^ Laboratoire de Génétique Moléculaire Service de médecine génétique CHUV Lausanne Switzerland

**Keywords:** Arrhythmia, autosomal recessive, long QT syndrome, Saudi Arabia

## Abstract

**Background:**

One of the most common primary cardiac arrhythmia syndromes is autosomal dominant long QT syndrome, type 1 (LQT1), chiefly caused by mono‐allelic mutations in the *KCNQ1* gene. Bi‐allelic mutations in the *KCNQ1* gene are causal to Jervell and Lange‐Nielsen syndrome (JLNS), characterized by severe and early‐onset arrhythmias with prolonged QTc interval on surface ECG and sensorineural deafness. Occasionally, bi‐allelic mutations in *KCNQ1* are also found in patients without any deafness, referred to as autosomal recessive long QT syndrome, type 1 (AR LQT1).

**Methods:**

We used Sanger sequencing to detect the pathogenic mutations in *KCNQ1* gene in eight families from Saudi Arabia with autosomal recessive LQT1.

**Results:**

We have detected pathogenic mutations in all eight families, two of the mutations are founder mutations, which are c.387‐5T>A and p.Val172Met/p.Arg293Cys (*in cis*). QTc and cardiac phenotype was found to be pronounced in all the probands comparable to the cardiac phenotype in JLNS patients. Heterozygous carriers for these mutations did not exhibit any clinical phenotype, but a significant number of them have sinus bradycardia.

**Conclusion:**

To the best of our knowledge, this is the first description of a large series of patients with familial autosomal recessive LQT, type 1. These mutations could be used for targeted screening in cardiac arrhythmia patients in Saudi Arabia and in people of Arabic ancestry.

**What's new?**

- Largest comprehensive phenotype–genotype study on eight families that have homozygous mutations in the KCNQ1 gene without having any auditory problem in the proband and so called Autosomal Recessive Long QT Syndrome, Type 1 (AR LQT1).
- Patients in five of the eight families carry the same mutation (c.387‐5T>A), which is considered founder mutations causal to AR LQT1. Second founder mutation is p.Val172Met/p.Arg293Cys (in cis)
- High rate of consanguinity could bring two identical autosomal dominant LQTS causal mutations together, resulting in AR LQT1.
- We also observed that heterozygous carriers for these mutations do not exhibit any clinical phenotype, but a significant number of them have sinus bradycardia, which we postulate could lead to the detection of a long QT syndrome family.

## Introduction

Congenital LQTS is an inherited cardiac repolarization disorder characterized by prolongation of corrected QT interval on the ECG and some low cardiac output events like syncope, seizures, and sudden cardiac death (SCD) due to ventricular tachyarrhythmia (Mizusawa et al. 2014). The most common form of LQTS is LQT1, with a prevalence of 1 in 2000 people worldwide (Mizusawa et al. 2014), which is inherited as an autosomal dominant pattern, while Jervell and Lange‐Nielsen syndrome (JLNS) is a rare condition observed in less than one per four million individuals (Mizusawa et al. 2014), which is an autosomal recessive disease, characterized by prolonged QTc, cardiac arrhythmias, and congenital deafness (Mizusawa et al. 2014). The 15 different genes identified so far (Mizusawa et al. 2014) associated with LQTS, encode for cardiac ion channels and its chaperones and are involved in the cardiac depolarization and repolarization. Among them most common are LQT1, LQT2, and LQT3, caused by mutations in *KCNQ1*,* KCNH2*, and *SCN5A* genes, respectively (Splawski et al. 2000; Westenskow et al. 2004; Mizusawa et al. 2014). Mutations in these three genes are found in more than 90% of genotype‐positive patients (Splawski et al. 2000; Westenskow et al. 2004; Mizusawa et al. 2014). Among all the reported mutations, missense mutations constitute the major type followed by frameshift mutations, in‐frame deletions, nonsense and splice site mutations (Splawski et al. 2000; Westenskow et al. 2004; Mizusawa et al. 2014).

*KCNQ1* gene encodes for the Kv7.1 subunit (potassium channel) of the slowly activating delayed rectifier outward K^+^ current (I_Ks_) channel, found in the heart and inner ear (Bhuiyan and Wilde 2013). Heterozygous nonsense or truncating mutations in *KCNQ1* lead to loss of function of the mutant allele (haploinsufficiency), which usually could not exert any dominant negative effect on the opposite normal allele (Bhuiyan and Wilde 2013). Hence, mono‐allelic *KCNQ1* nonsense or truncating mutation carriers are often asymptomatic (Bhuiyan and Wilde 2013). But, heterozygous missense mutations with its dominant negative effect on the opposite normal allele confer pathogenicity and lead to autosomal dominant LQT1 and its sequelae (Bhuiyan and Wilde 2013). Homozygous or compound heterozygous mutations that lead to complete loss of function of the I_Ks_ protein are causal to the recessive form of the disease, Jervell and Lange‐Nielsen syndrome (JLNS), with more severe cardiac phenotype than the dominant form of LQT1 and with the additional feature of Sensorineural Hearing Loss (SNHL) (Bhuiyan and Wilde 2013).

Occasionally, with homozygous or compound heterozygous mutations in the *KCNQ1* gene, there could still remain some I_Ks_ protein residues (Bhuiyan and Wilde 2013). These individuals present cardiac manifestations only, without any hearing defect; this is regarded as autosomal recessive LQTS type 1 (AR LQT1), which was first reported by Priori et al. in 1998 (5). Since then, few other studies have been published describing this type of AR LQT1 (Priori et al. 1998; Larsen et al. 1999; Ning et al. 2003; Westenskow et al. 2004; Novotny et al. 2006; Bhuiyan et al. 2008; Jackson et al. 2014; Vyas et al. 2016).

The first report on AR LQT1 in Saudi Arabia was published in 2008 (Bhuiyan et al. 2008). Bhuiyan et al. (2008) described a novel homozygous founder mutation in the splice site at intron‐1‐exon‐2 of the *KCNQ1* gene (c.387‐5T>A) which caused only severe arrhythmias (without any auditory phenotype) in three children from two families (Bhuiyan et al. 2008). Most cases of AR LQT1 reported so far have been sporadic affecting a single individual. In this prospective study, we present our data from eight families in Saudi Arabia that have homozygous mutations in the *KCNQ1* gene without having any auditory problem in the proband and in their siblings. To the best of our knowledge, this is the first description of a large series of patients with familial AR LQT1.

## Methodology

### Clinical analysis

Eight families with a family history of syncope, seizures, and sudden cardiac death were diagnosed by the cardiologists and recruited for genetic study. Clinical investigation was performed in the eight probands and their families by taking full family history, ECG, and construction of family pedigrees. Families involved in this study are not related, all originate from different regions in Saudi Arabia. All patients with homozygous mutations underwent hearing tests, resting ECG was also performed. ECG was also performed in heterozygous mutation carriers. Hearing test was not performed in heterozygous mutation carriers if there was no indication for any hearing defect.

### Mutation analysis

Molecular genetic investigation was done at Princess Al jawhara Center of Excellence in Research of Hereditary Disorders (PACER‐HD), King Abdulaziz University, Jeddah, Saudi Arabia. Blood samples were collected and the DNA was isolated from peripheral blood leukocytes using DNA blood mini kit (QIAmp, Qiagen, Inc., Hilden, Germany) following the manufacturer's instructions. All 16 exons of the *KCNQ1* genes (RefSeq: NM_000218, UniProt P51787, OMIM 607542, LRG_287) were amplified from genomic DNA by primers that cover all exons and exon intron boundaries, the primers were designed using web‐based primer design software, Primer3Plus http://www.bioinformatics.nl/cgi-bin/primer3plus/primer3plus.cgi/.

PCR products were purified using QIAquick PCR purification kit then bidirectionally sequenced using a Big Dye Terminator v3.1Cycle Sequencing Kit on an ABI 3500 sequencer (Thermo Fisher Scientific Inc, Applied Biosytems, Foster City, CA, USA). Sequence variants were analyzed via BioEdit sequence alignment editor version 6.0.7 (http://www.mbio.ncsu.edu/bioedit/bioedit.html).

## Results

### Family A

**Figure 1:**
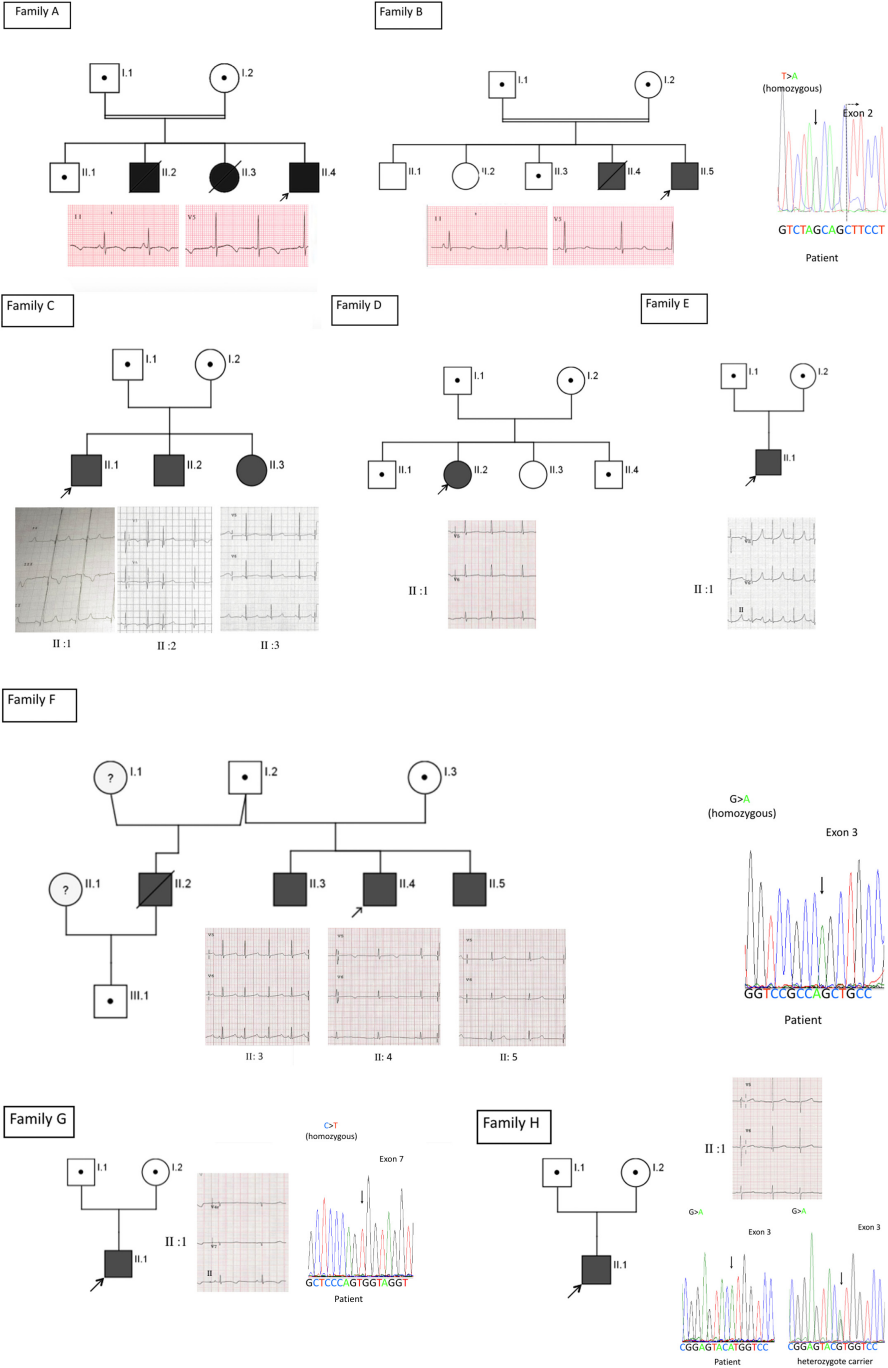
Pedigrees structure of eight families with AR LQT1 patients. Proband in each family is shown with an arrow mark. Circles are denoted for female and squares are denoted for male. Black‐filled circles and squares in all pedigrees are confirmed AR LQT1 patients. Half‐filled circles or squares denote heterozygotes for the familial mutation. Consanguineous marriages are indicated by =. Deceased individuals with the disease are shown with a diagonal line. Baseline ECG from the probands and all homozygote family members are shown below or beside each family tree.

The proband (II: 4 in Fig. 1) was a 3‐year‐old boy who was suspected to have epilepsy after he experienced an episode of syncope while swimming. After his neurological evaluation was found to be normal, he was diagnosed as LQTS with QTc = 557 ms (Table 1). His syncopal attacks never recurred after he was started on Atelonol 12.5 mg/day. He had a brother and sister (II:2 and 3 in Fig. 1) who died suddenly (at the age of 10 years while diving and 5 years while at the playground, respectively). The sister had a history of loss of consciousness and was diagnosed with LQTS but not treated. All the children had normal hearing tests. The parents (I:1 and 2 in Fig. 1) were consanguineous, asymptomatic, and had normal ECGs. The proband (II:4 in Fig. 1 and Table 1) was found to be homozygous for the mutation in intron 1 of *KCNQ1* gene at 5‐base upstream from the first nucleotide of exon‐2 (c.387‐5 T>A; NCBI Ref. NM_000218) and had been reported as rs794728549.

**Table 1:** Genotype status of all the genotype‐positive patients from the eight families with AR LQT1 patients. Clinical symptoms (if any), QTc interval, pharmacological intervention (if any), and other relevant parameters of the AR LQT1 patients as well as the heterozygote family members are also mentioned

Family numbers	ID, age(yrs), gender	Age at first presentations (yrs)	Symptoms	QTc (ms)	Genotype	Medication	Response to medication
A	II:4, 3, m		Syncope	557	c.[387‐5 T>A]; [387‐5 T>A] p. [?]; [?]	Atenolol (12.5 mg bd)	Well response
	II:1, m		n.s	430	c.[387‐5 T>A]; [=] p. [?]; [=]	n.m	
	I:1, m		n.s	459	c.[387‐5 T>A]; [=] p. [?]; [=]	n.m	
	I:2, f		n.s	431	c.[387‐5 T>A]; [=] p. [?]; [=]	n.m	
B	II:5, 16, m	1	Seizure attacks	529	c.[387‐5 T>A]; [387‐5 T>A] p. [?]; [?]	Propranolol (20 mg tid)	Well response
	II:3, m		n.s	n.a	c.[387‐5 T>A]; [=] p. [?]; [?]	n.m	
	I:1, m		n.s	n.a	c.[387‐5 T>A]; [=] p. [?]; [=]	n.m	
C	II:1, 16, m			500	c.[387‐5 T>A]; [387‐5 T>A] p. [?]; [ ?]		
	II:2, 9, m		Dizziness	459	c.[387‐5 T>A]; [387‐5 T>A] p. [?]; [?]	Inderal	
	II:3, 8, f		n.s	498	c.[387‐5 T>A]; [387‐5 T>A] p. [?]; [?]	Inderal	
	I:1, 44, m		n.s	444	c.[387‐5 T>A]; [=] p. [?]; [=]	n.m	
	I:2, 44, f		n.s	467	c.[387‐5 T>A]; [=] p. [?]; [=]	n.m	
D	II:2,9, f		Episode of cardiac arrest	537	c.[387‐5 T>A]; [387‐5 T>A] p. [?]; [?]	Propranolol (20 mg tds),	
	II:1, 15, m		Bradycardia	435	c.[387‐5 T>A]; [=] p. [?]; [=]	n.m	
	II:4, 2, f		n.s	474	c.[387‐5 T>A]; [=] p. [?]; [=]	n.m	
	I:1, m		Bradycardia	404	c.[387‐5 T>A]; [=] p. [?]; [=]	n.m	
	I :2, f		Bradycardia	457	c.[387‐5 T>A]; [=] p. [?]; [=]	n.m	
E	II:1, 1, f	Since birth	Bradycardia	531	c.[387‐5 T>A]; [387‐5 T>A] p. [?]; [?]	Propranolol (1 mg/kg)	Well response
	I:1, 32, m		n.s	399	c.[387‐5 T>A]; [=] p. [?]; [=]	n.m	
	I:2, 25, f		Bradycardia	418	c.[387‐5 T>A]; [=] p. [?]; [=]	n.m	
F	II:4, 12, m	3	Palpitation, seizures, chest pain, loss of consciousness	534	c.[535G>A]; [535G>A] p.[Gly179Ser]; [Gly179Ser]	B. blocker (2 mg/kg/bd)	Well response
	II:3, 16, m		n.s	444	c.[535G>A]; [535G>A] p.[Gly179Ser]; [Gly179Ser]	B. blocker (2 mg/kg/bd)	
	II:5, 8 mos, m		n.s	496	c.[535G>A]; [535G>A] p.[Gly179Ser]; [Gly179Ser]	B. blocker (2 mg/kg/bd)	
	III:1, 6, m		n.s	448	c.[535G>A]; [=] p.[Gly179Ser]; [=]	ICD implanted	
	I:2, 60, m		n.s	467	c.[535G>A]; [=] p.[Gly179Ser]; [=]	n.m	
G	II:1, 7, m		Syncope, bradycardia	580	c.[1031 C>T]; [1031 C>T] p.[A344V]; [A344V]		
	I:1,, m		n.s	443	c.[1031 C>T]; [=] p.[A344V]; [=]	n.m	
	I:2,, f		n.s	478	c.[1031 C>T]; [=] p.[A344V]; [=]	n.m	
H	II:1,12, m	11	Syncope	485	c.[514G>A]; [514G>A] p. [V172M];[ V172M] and c.[877C>T]; [877C>T] p. [R293C];[R293C]	Propranolol (10 mg tid)	
	I:1,44, m		n.s	417	c.[514G>A; 877C>T]; [=] p.[V172M; R293C];[ =]	n.m	
	I:2, 42, f		n.s	415	c.[514G>A; 877C>T]; [=] p. [V172M; R293C];[ =]	n.m	

n.a, not available; n.s, no symptoms; n.m, no medication.

Moreover, the analysis showed that his parents (I:1 and I:2 in Fig. 1 and Table 1) and a living brother (II:1 in Fig. 1 and Table 1) were heterozygous for the same mutation. Detailed clinical phenotype and in vitro electrophysiology analysis data on this family was published previously (Bhuiyan et al. 2008).

### Family B

The proband (II: 5 in Fig. 1) was a 16‐year‐old boy who developed generalized seizures at the age of 1 year, lasting 1–2 min with cyanosis of the lips. The attacks were triggered by physical activity and uncontrolled by anti‐epileptics. At the age of 4 years, he was reevaluated and diagnosed with LQTS with prolonged QT interval. His seizure attacks stopped after starting propranolol treatment. At the age of 16, he was on 20 mg propranolol three times per day and playing sports without problems. His ECG showed prolonged QTc of 529 ms (Table 1). His hearing tests were normal. There is a family history of seizures in an older brother who died at the age of 2 years (II: 4 in Fig. 1), who had no hearing abnormality. His parents and other siblings were asymptomatic (I1, 2 and II: 1,2 and 3 in Fig. 1). Genetic investigation detected homozygous mutation c.387‐5T>A in the *KCNQ1* gene in II: 5 (Fig. 1 and Table 1). This was found heterozygously in his father and one brother (I:1 and II: 3 in Fig. 1 and Table 1). The mother's DNA was not available for genetic testing. Detailed clinical phenotype and functional data on this family was published previously (Bhuiyan et al. 2008).

### Family C

The proband (II:1 in Fig. 1) was a 16‐year‐old boy who had syncope at the age of 10 years, referred to cardiology clinic and diagnosed with LQTS. He was diagnosed previously to have seizure disorder at the age of 8 months. He is on propranolol treatment. His 9‐year‐old brother (II:2 in Fig. 1) only had dizziness and is on propranolol. An 8‐year‐old sister (II:3 in Fig. 1) is asymptomatic, her heart rate was 79 beats/min, she was also prescribed Inderal. All three siblings had sinus rhythm and prolonged QTc (500 ms, 459 ms, 498 ms, respectively) (Table 1). Their hearing tests were normal. Their parents (I:1 and I:2 in Fig. 1) were both asymptomatic with normal ECGs and no hearing defect. Genetic testing showed the three children (II: 1, 2, and 3 in Fig. 1 and Table 1) hosting homozygous mutations c.387‐5T>A in the exon‐2/intron‐1 junction of the *KCNQ1* gene. Both parents were heterozygous (I:1 and I:2 in Fig. 1 and Table 1) for the same mutation.

### Family D

The proband (II: 2 in Fig. 1) was a 9‐year‐old girl who had a single 10‐min episode of cardiac arrest that responded to CPR. She was diagnosed with LQT1 and ICD was advised but the family refused. She is on propranolol 20 mg TDS. She has a strong family history of sudden death (four members), Her ECG showed sinus rhythm (80 beats/min) and marked QT prolongation (537 ms) (Table 1). ECG from the father, mother, and one brother (I :1,2 and II:1 in Fig. 1) showed bradycardia (46, 52, 47 beats/min, respectively). Another brother (II:4 in Fig. 1) had prolonged QTc intervals (474 ms) (Table 1). All the children including the proband had normal hearing tests. The proband (II:2 in Fig. 1 and Table 1) was found to have homozygous mutation c.387‐5T>A in the exon‐2/intron‐1 junction of the *KCNQ1* gene. Three family members (I:1, 2 and II:1, 4 in Fig. 1 and Table 1) were heterozygous carriers for the same mutation.

### Family E

The proband (II: 1 in Fig. 1) was a 1‐year‐old girl who had bradycardia since birth and congenital heart defect (venticular septal defect which closed spontaneously). The ECG revealed a prolonged QTc interval. She does not have SNHL. There is no family history of sudden death, arrhythmia, or SNHL. She is currently on propranolol 1 mg/kg. Her latest ECG showed sinus rhythm (115 beats/min) and marked QTc prolongation (531 msec) (Table 1). Her parents had normal ECGs (Table 1). The proband (II: 1 in Fig. 1 and Table 1) was found to have a homozygous mutation c.387‐5T>A in the *KCNQ1* gene. Her parents (I:1 and I:2 in Fig. 1 and Table 1) were heterozygous for the same mutation.

### Family F

The proband (II: 4 in Fig. 1) was a 12‐year‐old boy who had palpitations and chest pain starting at the age of 3 years related to fear and auditory stimuli. He had recurrent loss of consciousness 10–15 min per episode, approximately three times per day. He was initially diagnosed as having epileptic seizures and prescribed anti‐epileptic medication without any improvement. Recently, he was diagnosed with LQTS with a QTc interval of 534 ms. He was started on propranolol and the symptoms improved drastically. His parents are asymptomatic. He has two asymptomatic brothers, 16 years old and 8 months old (II: 3 and 5 in Fig. 1). The elder brother's QTc interval was 444 ms (upper limit of normal). The younger brother had prolonged QTc interval (496 ms) (Table 1). All the children including the proband had normal hearing tests. There is a history of seizures in a paternal half‐sister and sudden death in a 24‐year‐old paternal half‐brother (II: 2 in Fig. 1). This brother has a child who is now 6 years old (III:1 in Fig. 1). There is a family history of sudden death and suspected LQTS in extended members of the proband's father's family. Also, the proband's maternal aunt has deafness. Genetic testing showed homozygous mutations c.535G>A (p.Gly179Ser) in the proband and his two siblings (II: 3, 4 and 5 in Fig. 1 and Table 1). Their father and the 6‐year‐old nephew (I:2 and III:1 in Fig. 1 and Table 1) were found heterozygous carriers for the same mutation. Mother declined to do the genetic testing.

### Family G

The proband (II: 1 in Fig. 1) was a 7‐year‐old boy who had one episode of syncope precipitated by fear. His hearing tests were normal. There is a family history of sudden deaths in two siblings, first one at the age of 4 years with the diagnosis of seizure disorder, while the other one died at the age of 1 year after a short febrile illness. The proband's ECG showed sinus bradycardia (57 beats/min) and prolonged QTc (580 ms) (Table 1). The ECGs of the parents were also abnormal showing prolonged QTc (Table 1). The proband (II: 1 in Fig. 1 and Table 1) was found to have homozygous mutations c.1031C>T (p.Ala344Val) in the *KCNQ1* gene. His parents (I:1 and I:2 in Fig. 1 and in Table 1) were heterozygous for the same mutation.

### Family H

The proband (II: 1 in Fig. 1) is a 12‐year‐old boy who experienced one syncopal attack at the age of 11 years. The ECG showed prolonged QTc intervals and was started on propranolol. Since then there had been no events of syncope, loss of consciousness, or seizures. His hearing tests were normal. There is no family history of LQTS. The proband is currently on propranolol 10 mg TDS (3× per day). His ECG showed sinus rhythm (67 beats/min) and marked QTc prolongation (485 msec) (Table 1). The proband (II: 1 in Fig. 1 and Table 1) has two homozygous mutations c.514G>A (p.Val172Met) and c.877C>T (p.Arg293Cys) in the *KCNQ1* gene, both mutations are located *in cis*. His parents (I:1 and I:2 in Fig. 1 and in Table 1) were found to be heterozygous for both mutations in *cis*, c.[514G>A;877C>T], p.[Val172Met;Arg293Cys], that is, they are not compound heterozygous mutations.

### Bradycardia in the heterozygous mutation carriers

Bradycardia was occasionally reported in patients with clinically symptomatic autosomal dominant LQT1 patients with heterozygous KCNQ1 mutations (Moss et al. 1991; Lupoglazoff et al. 2004). We tried to assess the heart rate in nonsymptomatic, heterozygous, mild, *KCNQ1* mutation carriers. As patients with homozygous mutations were having *β*‐blockers, we evaluated the family members having a heterozygous mutation in the *KCNQ1* gene. In total, we have analyzed the heart rate in 16 heterozygous *KCNQ1* mutation carriers; sinus bradycardia (≤60 beats/min) was observed in 50% of the heterozygous carriers (*n* = 8), matching against age‐specific control values. This has been shown in Table 2.

**Table 2:** Basal heart rate of the heterozygous *KCNQ1* mutation carriers (without *β*‐blocker)

Family id	Patient's name	Mutation	Basal heart rate
Family A	Father of proband	c.387‐5T>A	53 beats/min
	Mother of proband	c.387‐5T>A	78 beats/min
Family B	Mother of proband	c.387‐5T>A	55 beats/min
Family C	Father of proband	c.387‐5T>A	75 beats/min
	Mother of proband	c.387‐5T>A	79 beats/min
Family D	Father of proband	c.387‐5T>A	58 beats/min
	Mother of proband	c.387‐5T>A	65 beats/min
	16‐year‐old brother of proband	c.387‐5T>A	60 beats/min
Family E	Father of Proband	c.387‐5T>A	72 beats/min
	Mother of proband	c.387‐5T>A	52 beats/min
Family F	Father of proband	p.Gly179Ser	79 beats/min
	Son of proband	p.Gly179Ser	60 beats/min
Family G	Father of proband	p.Ala344Val	65 beats/min
	Mother of proband	p.Ala344Val	54 beats/min
Family H	Father of proband	p.Arg293Cys	60 beats/min
	Mother of proband	p.Arg293Cys	83 beats/min

## Discussion

In this study, we performed comprehensive clinical and molecular screening of the *KCNQ1* gene in a series of patients from eight families with AR LQT1 in Saudi Arabia. Mutation c.387‐5T>A was detected in five families making it likely the most common LQT1 causal founder mutation in Saudi Arabia. Previously, two of these families (A and B) were reported by Bhuiyan et al. (10). Additional AR LQT1 causal mutations detected are: p.Gly179Ser, p.Ala344Val, p.Val172Met, p.Arg293Cys found in three more families. p.Val172Met and p.Arg293Cys are situated *in cis*. p.Gly179Ser mutation has been previously reported in the compound heterozygous situation with p.Gln530X in autosomal recessive LQT1 (Giudicessi and Ackerman 2013). The p.Ala344Val mutation has been described as a mild/forme fruste LQT1 mutation (Donger et al. 1997; Siebrands et al. 2006), it was also described in connection to increased local anesthetic sensitivity (Siebrands et al. 2006; Hedley et al. 2013). Later, Choi et al. (2004) discovered this mutation in a cohort of patients, who had suffered swimming‐triggered arrhythmias. p.Val172Met and p.Arg293Cys, located *in cis,* has first been described in Qatari population by exome sequencing (Yavarna et al. 2015). This is very likely another founder mutation in the Arab region, which deserves further population studies (Yavarna et al. 2015).

None of the probands with homozygous mutations in our study have deafness, but, their cardiac phenotypes are severer and the QTc is longer than patients with the autosomal dominant long QT syndrome (Priori et al. 1998; Larsen et al. 1999; Ning et al. 2003; Westenskow et al. 2004; Novotny et al. 2006; Bhuiyan et al. 2008; Jackson et al. 2014; Vyas et al. 2016). This is in agreement with the hypothesis drawn by Bhuiyan et al. (2008); Bhuiyan and Wilde 2013), and Priori et al. (Priori et al. 1998), which explains that KCNQ1 mutations causal to recessive LQT1 are usually mild and they could still have some residual I_Ks_ in the patient's heart and ear. On the other hand, JLNS causal KCNQ1 mutations are severe and I_Ks_ is completely absent in JLNS patients (Priori et al. 1998; Bhuiyan et al. 2008). This residual minor quantity of I_Ks_ is sufficient to preserve the hearing function in recessive LQT1 patients (Bhuiyan et al. 2008).

All the 16 heterozygous mutation carriers were asymptomatic. Their QTcs were normal to only slightly prolonged. However, eight of these mutation carriers had sinus bradycardia. This observation of sinus bradycardia is known to occur in heterozygous carriers of other arrhythmia‐related genes (SCN5A, HCN4 etc) (Milanesi et al. 2006; Selly et al. 2012; Milano et al. 2014). However, nothing was known about sinus bradycardia in *KCNQ1* mild mutation carriers, who does not have any clinical phenotype. Bradycardia has been previously reported in neonates with severe KCNQ1 mutations (Lupoglazoff et al. 2004). In our study, we did not observe any cardiac symptoms of LQT1 in the mild heterozygous *KCNQ1* mutation carriers. Such mild mutation carriers may go unnoticed for any possible therapeutic or behavioral intervention as the carriers lack any cardiac phenotype. Sinus bradycardia in a family member with sudden cardiac death or unexplained arrhythmia might lead a physician to consider about the possible carrier status for a *KCNQ1* mutation in the family. Another aspect could also be considered; such mild mutations are possibly harmless in normal situation, but, such carriers could have arrhythmia symptoms due to the use of QT prolonging drugs or under extreme physical or mental stress, by compromising the repolarization reserve.

In conclusion, we describe a series of patients with AR LQT1, all originating from consanguineous families in Saudi Arabia. These mutations could be used for targeted screening in cardiac arrhythmia patients in Saudi Arabia and people of Arab ancestry. This study further confirms that recessive LQT1 patients do not have any deafness but their QTc are still pronounced and cardiac symptoms are more severe than the patients with dominant form of LQT1. Carriers are likely to have bradycardia.

## Conflict of Interest

No conflict of interest declared by any authors of the manuscript.
